# Crystal structure of 3,13-dimethoxy-5,6,10,11-tetrahydrofuro[3,4-*i*][5]helicene-7,9-dione

**DOI:** 10.1107/S1600536814023137

**Published:** 2014-10-29

**Authors:** Thanasat Sooksimuang, Siriporn Kamtonwong, Waraporn Parnchan, Laongdao Kangkaew, Somboon Sahasithiwat

**Affiliations:** a National Metal and Materials Technology Center (MTEC), 114 Thailand Science, Park, Paholyothin Rd., Klong Luang, Pathumthani, 12120, Thailand

**Keywords:** crystal structure, helicene, hydrogen bonds

## Abstract

The title compound, C_26_H_20_O_5_, crystallizes with two independent molecules (*A* and *B*) in the asymmetric unit, which differ primarily in the location of the –OCH_3_ groups. In the crystal, the mol­ecules form a layered structure parallel to (10-1) by C—H⋯O hydrogen-bonded networks. Adjacent layers are also linked by further C—H⋯O hydrogen bonds, forming a three-dimensional structure.

## Abstract 2

In the crystal, the mol­ecules form a layered structure parallel to (10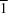) *via* C—H⋯O hydrogen-bonding inter­actions. Adjacent layers are also linked by C—H⋯O hydrogen bonds, forming a three-dimensional structure.

## Chemical context   

Helicenes are polycyclic aromatic hydro­carbons (PAHs) consisting of *ortho*-fused aromatic rings arranged in a helical chirality. Among various applications of helicenes (Shen & Chen, 2012 ▶; Gingras, 2013 ▶), the use of helicene derivatives as light emitters in organic light-emitting diodes has been reported (Sahasithiwat *et al.*, 2010 ▶; Shi *et al.*, 2012 ▶). The title compound is a derivative of penta­helicene in which two electron-donating groups, *i.e.* meth­oxy –OCH_3_, and an electron-withdrawing group, *i.e.* di­carb­oxy­lic anhydride –C(=O)OC(=O)–, are added onto the structure. The arrangement of electron donating and withdrawing groups are set into a Λ-shape with the electron-withdrawing group located in the middle, resulting in an effective push–pull system. Moreover, The two rings connected to the central benzene ring are non-aromatic and are in a twist conformation.
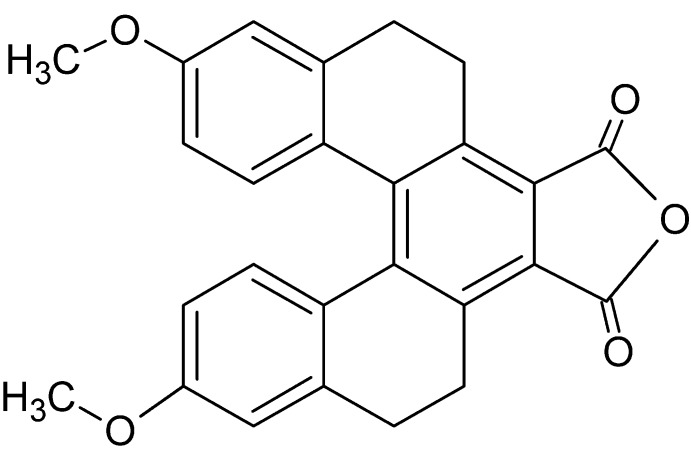


## Structural commentary   

**Figure 1:**
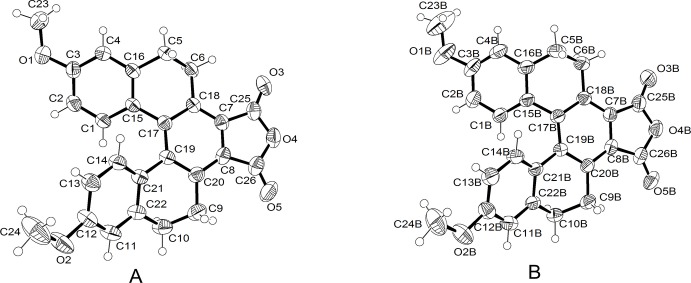
Mol­ecular structure of mol­ecules *A* and *B* of the title compound showing 50% probability displacement ellipsoids for non-H atoms.

**Figure 2:**
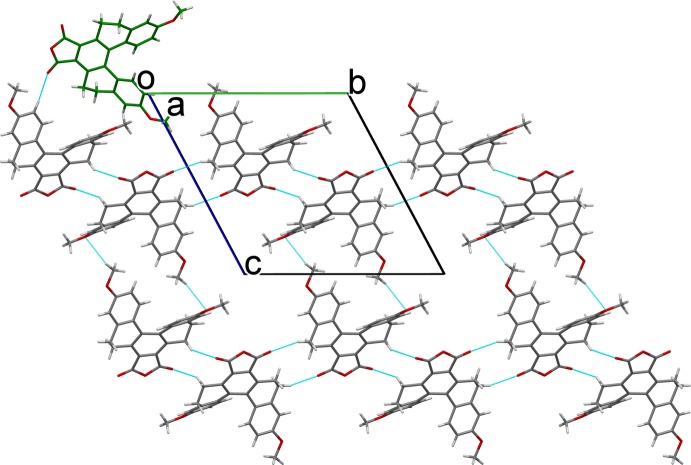
Part of the crystal structure, projected along the *a* axis, depicting a layer consisting of *B* mol­ecules linked through hydrogen bonds (blue dashed lines) and connecting to *A* mol­ecules by further hydrogen bonds. The carbon atoms of mol­ecules *A* (green) and *B* (dark gray) are colored differently.

The geometric parameters of the title mol­ecule agree well with those reported for similar structures (McIntosh *et al.*, 1954 ▶; Wang *et al.*, 1997 ▶; Stammel *et al.*, 1999 ▶; Rajapakse *et al.*, 2011 ▶). The asymmetric unit of the title compound contains two independent mol­ecules (*A* and *B*), as shown in Fig. 1 ▶. The title compound crystallizes as a racemate in the space group *P* 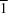. The enanti­omeric (*P*)-form is the mirror geometry of the (*M*)-form. The torsion angle along the inner helical rim of mol­ecule *A* [C15—C17—C19—C21, −20.3 (3)°] differs from that of mol­ecule *B* [C15*B*—C17*B*—C19*B*—C21*B*, 24.8 (3)°] primarily as a result of the differences in the location of their meth­oxy groups. Also, the torsion angles between a terminal ring and a meth­oxy group of mol­ecule *A* and *B* of the same form are significantly different, *e.g.* C24—O2—C12—C13 [−2.9 (4)°] *vs* C24*B*—O2*B*—C12*B*—C13*B* [−5.7 (4)] and C23—O1—C3—C2 [170.0 (2)] *vs* C23*B*—O1*B*—C3*B*—C2*B* [−176.9 (3)]. Moreover, unlike in another 3,12-dimeth­oxy[5]helicene derivative (Sahasithiwat *et al.*, 2014 ▶) where both meth­oxy groups are bent inward, one of meth­oxy groups of the title compound is bent outward. In mol­ecule *A*, this outward bending results from C23—H23*A*⋯O3*B*(*x* − 1, *y* − 1, *z*) hydrogen bonding, while in mol­ecule *B*, the bending is the result of steric hindrance between atoms C23*B* and C24*B*(−*x* + 1, −*y* + 1, −*z*) of paired mol­ecules (Fig. 2 ▶).

## Supra­molecular features   

**Figure 3:**
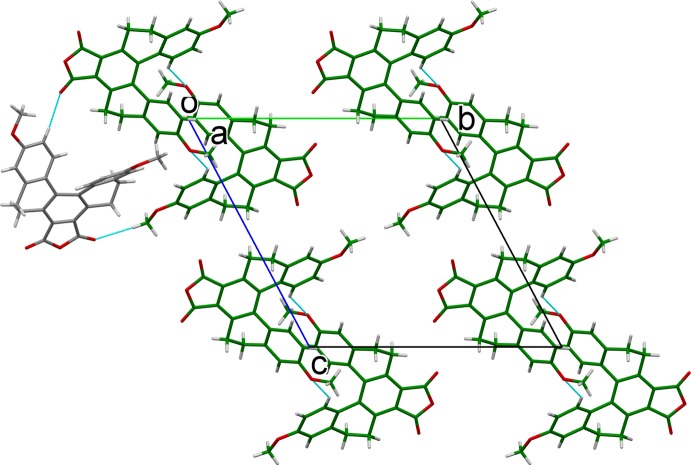
Part of the crystal structure, projected along the *a* axis, depicting a layer consisting of *A* mol­ecules linked through hydrogen bonds (blue dashed lines) and connecting to *B* mol­ecules by further hydrogen bonds. The carbon atoms of mol­ecules *A* (green) and *B* (dark gray) are colored differently.

**Figure 4:**
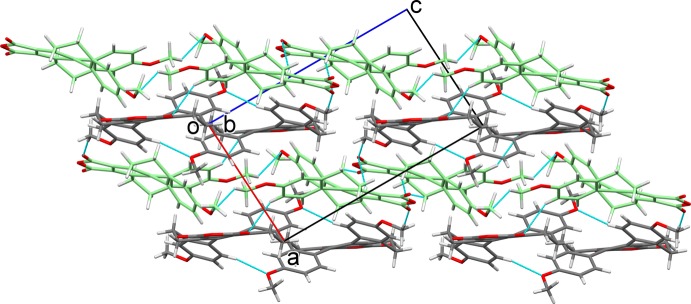
Packing of the crystal structure, projected along the *b* axis, showing the layered structure. The carbon atoms of mol­ecule *A* (green) and *B* (dark gray) are colored differently.

In the crystal structure, C—H⋯O hydrogen-bonding inter­actions (Table 1 ▶) between *B* mol­ecules leads to a formation of a mol­ecule *B* layer (Fig. 2 ▶), while C—H⋯O hydrogen-bonding inter­actions involving *A* mol­ecules leads to the formation of a mol­ecule *A* layer (Fig. 3 ▶). The two layers are positioned alternately parallel to (10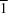), as displayed in Fig. 4 ▶. Adjacent layers are connected by further C—H⋯O hydrogen bonds, forming a three-dimensional structure.

## Synthesis and crystallization   

The diene 6,6′-dimeth­oxy-3,4,3′,4′-tetra­hydro­[1,1′]bi­naphthalenyl (48 g, 0.15 mol), maleic anhydride (75 g, 0.76 mol) and toluene (65 ml) were place in a 250 ml round-bottom flask and the reaction mixture was stirred at room temperature under an argon atmosphere for 5 days. The resulting mixture was poured into water (300 ml) with vigorous stirring. The organic layer was separated and the aqueous layer was extracted with ethyl acetate (3 x 100 ml). The combined organic layer was dried with Na_2_SO_4_ and the organic solvents were removed to yield a Diels–Alder adduct. The crude product was purified by column chromatography (silica gel, ethyl acetate–hexa­ne) to give the inter­mediate compound (31.67 g, 51%) as a yellow viscous liquid. The Diels–Alder adduct (31 g, 0.07 mol), 2,3-di­chloro-5,6-di­cyano-1,4-benzo­quinone (DDQ) (34 g, 0.15 mol) and xylene (500 ml) were mixed and refluxed for 8 h under an argon atmosphere. The reaction mixture was allowed to cool to room temperature, filtered, and the solid was washed with di­chloro­methane (600 ml). The solvents were removed from the filtrate under reduce pressure to gain the crude product, which was further purified by column chromatography (silica gel, ethyl acetate–hexa­ne) to give the title compound (18.3g, 60%) as a yellow solid, which was characterized by FTIR, ^1^H-NMR and ^13^C-NMR. Crystals suitable for X-ray analysis were obtained by slow vapor diffusion of hexane into a solution of the title compound in chloro­form.

### Refinement   

Crystal data, data collection and structure refinement details are summarized in Table 2 ▶. All hydrogen atoms were placed in calculated positions and treated as riding atoms with C—H = 0.93–0.97 Å and with *U*_iso_ = 1.5*U*_eq_(C) for methyl H atoms and 1.2*U*_eq_(C) for other H atoms.

## Supplementary Material

Crystal structure: contains datablock(s) I, New_Global_Publ_Block. DOI: 10.1107/S1600536814023137/nr2055sup1.cif

Structure factors: contains datablock(s) I. DOI: 10.1107/S1600536814023137/nr2055Isup2.hkl

Supporting information file. DOI: 10.1107/S1600536814023137/nr2055Isup4.mol

CCDC reference: 1030212

Additional supporting information:  crystallographic information; 3D view; checkCIF report

## Figures and Tables

**Table 1:** Hydrogen-bond geometry (, )

*D*H*A*	*D*H	H*A*	*D* *A*	*D*H*A*
C1H1O2^i^	0.93	2.58	3.304(3)	135
C23H23*A*O3*B* ^ii^	0.96	2.71	3.666(3)	177
C6*B*H6*D*O3*B* ^iii^	0.97	2.68	3.547(3)	149
C10*B*H10*C*O5*B* ^iv^	0.97	2.44	3.297(3)	147
C13*B*H13*B*O5^v^	0.93	2.56	3.408(3)	152
C24*B*H24*D*O1*B* ^v^	0.96	2.74	3.491(4)	136

Symmetry codes: (i) 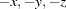; (ii) 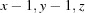; (iii) 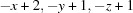; (iv) 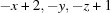; (v) 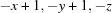.

**Table 2:** Experimental details

Crystal data	
Chemical formula	C_26_H_20_O_5_
*M*_r_	412.42
Crystal system, space group	Triclinic, *P* 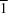
Temperature (K)	296
*a*, *b*, *c* ()	8.7570(9), 15.9008(16), 16.2987(16)
, , ()	61.695(3), 84.535(3), 84.460(3)
*V* (^3^)	1985.6(3)
*Z*	4
Radiation type	Mo *K*
(mm^1^)	0.10
Crystal size (mm)	0.42 0.34 0.16

Data collection	
Diffractometer	Bruker APEXII CCD
Absorption correction	Multi-scan (*SADABS*; Bruker, 2012 ▶)
*T*_min_, *T*_max_	0.67, 0.75
No. of measured, independent and observed [*I* > 2(*I*)] reflections	33986, 7072, 4457
*R*_int_	0.049
(sin /)_max_ (^1^)	0.597

Refinement	
*R*[*F* ^2^ > 2(*F* ^2^)], *wR*(*F* ^2^), *S*	0.049, 0.140, 1.02
No. of reflections	7072
No. of parameters	559
H-atom treatment	H-atom parameters constrained
_max_, _min_ (e ^3^)	0.55, 0.29

Computer programs: *APEX2* (Bruker, 2008 ▶), *SAINT* (Bruker, 2013 ▶), *SHELXS2013* and *SHELXL2013* (Sheldrick, 2008 ▶), *ORTEP-3 for Windows* and *WinGX* (Farrugia, 2012 ▶), *Mercury* (Macrae *et al.*, 2006 ▶) and *publCIF* (Westrip, 2010 ▶).

## supplementary crystallographic information

### Crystal data

**Table:**

C_26_H_20_O_5_	*Z* = 4
*M**_r_* = 412.42	*F*(000) = 864
Triclinic, *P*1	*D*_x_ = 1.380 Mg m^−^^3^
*a* = 8.7570 (9) Å	Mo *K*α radiation, λ = 0.71073 Å
*b* = 15.9008 (16) Å	Cell parameters from 5829 reflections
*c* = 16.2987 (16) Å	θ = 2.3–21.3°
α = 61.695 (3)°	µ = 0.10 mm^−^^1^
β = 84.535 (3)°	*T* = 296 K
γ = 84.460 (3)°	Block, yellow
*V* = 1985.6 (3) Å^3^	0.42 × 0.34 × 0.16 mm

### Data collection

**Table:**

Bruker APEXII CCD diffractometer	4457 reflections with *I* > 2σ(*I*)
Radiation source: sealed tube	*R*_int_ = 0.049
φ and ω scans	θ_max_ = 25.1°, θ_min_ = 2.5°
Absorption correction: multi-scan (*SADABS*; Bruker, 2012)	*h* = −10→10
*T*_min_ = 0.67, *T*_max_ = 0.75	*k* = −18→18
33986 measured reflections	*l* = −18→19
7072 independent reflections	

### Refinement

**Table:**

Refinement on *F*^2^	0 restraints
Least-squares matrix: full	Hydrogen site location: inferred from neighbouring sites
*R*[*F*^2^ > 2σ(*F*^2^)] = 0.049	H-atom parameters constrained
*wR*(*F*^2^) = 0.140	*w* = 1/[σ^2^(*F*_o_^2^) + (0.0675*P*)^2^ + 0.2937*P*] where *P* = (*F*_o_^2^ + 2*F*_c_^2^)/3
*S* = 1.02	(Δ/σ)_max_ < 0.001
7072 reflections	Δρ_max_ = 0.55 e Å^−^^3^
559 parameters	Δρ_min_ = −0.29 e Å^−^^3^

### Special details

**Table:**

Geometry. All e.s.d.'s (except the e.s.d. in the dihedral angle between two l.s. planes) are estimated using the full covariance matrix. The cell e.s.d.'s are taken into account individually in the estimation of e.s.d.'s in distances, angles and torsion angles; correlations between e.s.d.'s in cell parameters are only used when they are defined by crystal symmetry. An approximate (isotropic) treatment of cell e.s.d.'s is used for estimating e.s.d.'s involving l.s. planes.

### Fractional atomic coordinates and isotropic or equivalent isotropic displacement parameters (Å^2^)

**Table:**

	*x*	*y*	*z*	*U*_iso_*/*U*_eq_	
O1	0.2293 (2)	−0.31891 (12)	0.40355 (12)	0.0598 (5)	
O2	0.1461 (2)	0.05931 (15)	−0.14110 (13)	0.0735 (6)	
O3	0.4061 (2)	0.25200 (12)	0.37700 (13)	0.0591 (5)	
O4	0.2984 (2)	0.36801 (12)	0.24856 (13)	0.0593 (5)	
O5	0.1804 (3)	0.45029 (13)	0.11326 (14)	0.0781 (6)	
C1	0.1382 (3)	−0.06644 (16)	0.26419 (16)	0.0426 (6)	
H1	0.0655	−0.0281	0.2217	0.051*	
C2	0.1326 (3)	−0.16423 (17)	0.30604 (16)	0.0467 (6)	
H2	0.0559	−0.1912	0.2921	0.056*	
C3	0.2405 (3)	−0.22305 (16)	0.36894 (16)	0.0446 (6)	
C4	0.3497 (3)	−0.18195 (16)	0.39277 (15)	0.0443 (6)	
H4	0.4207	−0.2208	0.4362	0.053*	
C5	0.4635 (3)	−0.03684 (16)	0.38211 (16)	0.0425 (6)	
H5A	0.5507	−0.0160	0.3372	0.051*	
H5B	0.5012	−0.0827	0.4423	0.051*	
C6	0.3807 (3)	0.04838 (16)	0.38856 (15)	0.0429 (6)	
H6A	0.3001	0.0263	0.4379	0.051*	
H6B	0.4526	0.0807	0.4042	0.051*	
C7	0.2975 (2)	0.21484 (16)	0.26582 (15)	0.0400 (5)	
C8	0.2294 (3)	0.27700 (16)	0.18311 (16)	0.0442 (6)	
C9	0.1102 (3)	0.31073 (17)	0.03254 (17)	0.0557 (7)	
H9A	0.1894	0.3356	−0.0174	0.067*	
H9B	0.0542	0.3644	0.0368	0.067*	
C10	0.0011 (3)	0.25601 (18)	0.01103 (18)	0.0552 (7)	
H10A	−0.0823	0.2348	0.0587	0.066*	
H10B	−0.0425	0.2972	−0.0487	0.066*	
C11	0.0742 (3)	0.14675 (18)	−0.06160 (16)	0.0517 (6)	
H11	0.0009	0.1797	−0.1055	0.062*	
C12	0.1672 (3)	0.07429 (18)	−0.06706 (16)	0.0499 (6)	
C13	0.2755 (3)	0.02482 (18)	−0.00206 (16)	0.0489 (6)	
H13	0.3399	−0.0233	−0.0061	0.059*	
C14	0.2879 (3)	0.04721 (16)	0.06923 (16)	0.0439 (6)	
H14	0.3613	0.0136	0.1129	0.053*	
C15	0.2512 (2)	−0.02339 (15)	0.28425 (15)	0.0375 (5)	
C16	0.3536 (2)	−0.08296 (16)	0.35214 (15)	0.0381 (5)	
C17	0.2585 (2)	0.08169 (15)	0.24154 (15)	0.0372 (5)	
C18	0.3120 (2)	0.11728 (16)	0.29812 (15)	0.0379 (5)	
C19	0.2096 (2)	0.14721 (16)	0.15063 (15)	0.0383 (5)	
C20	0.1827 (3)	0.24554 (16)	0.12332 (15)	0.0434 (6)	
C21	0.1934 (2)	0.11857 (16)	0.07733 (15)	0.0399 (5)	
C22	0.0884 (3)	0.17124 (16)	0.00842 (15)	0.0441 (6)	
C23	0.3521 (3)	−0.38130 (18)	0.45629 (19)	0.0671 (8)	
H23A	0.3298	−0.4465	0.4770	0.101*	
H23B	0.3631	−0.3720	0.5094	0.101*	
H23C	0.4459	−0.3673	0.4179	0.101*	
C24	0.2461 (4)	−0.0086 (3)	−0.1551 (2)	0.0984 (12)	
H24A	0.2190	−0.0113	−0.2095	0.148*	
H24B	0.2371	−0.0704	−0.1016	0.148*	
H24C	0.3500	0.0098	−0.1638	0.148*	
C25	0.3434 (3)	0.27254 (18)	0.30762 (19)	0.0476 (6)	
C26	0.2271 (3)	0.37458 (19)	0.1722 (2)	0.0552 (7)	
O1B	0.4341 (3)	0.70645 (14)	0.20432 (14)	0.0874 (7)	
O2B	0.5797 (2)	0.27135 (14)	0.06882 (13)	0.0675 (5)	
O3B	1.2533 (2)	0.37305 (14)	0.53046 (14)	0.0757 (6)	
O4B	1.22652 (19)	0.22009 (12)	0.56595 (11)	0.0583 (5)	
O5B	1.1588 (2)	0.08717 (13)	0.56674 (13)	0.0664 (5)	
C1B	0.6213 (3)	0.47154 (17)	0.28976 (16)	0.0464 (6)	
H1B	0.5927	0.4099	0.3086	0.056*	
C2B	0.5121 (3)	0.54570 (18)	0.26048 (17)	0.0527 (7)	
H2B	0.4102	0.5345	0.2593	0.063*	
C3B	0.5538 (3)	0.63758 (18)	0.23254 (17)	0.0559 (7)	
C4B	0.7039 (3)	0.65447 (17)	0.23540 (17)	0.0556 (7)	
H4B	0.7309	0.7164	0.2166	0.067*	
C5B	0.9769 (3)	0.59131 (17)	0.27941 (19)	0.0616 (7)	
H5C	0.9848	0.6539	0.2747	0.074*	
H5D	1.0451	0.5866	0.2311	0.074*	
C6B	1.0240 (3)	0.51389 (16)	0.37485 (18)	0.0547 (7)	
H6C	1.1310	0.5187	0.3818	0.066*	
H6D	0.9627	0.5230	0.4231	0.066*	
C7B	1.0800 (3)	0.33363 (16)	0.44698 (15)	0.0412 (6)	
C8B	1.0537 (2)	0.24431 (16)	0.45687 (15)	0.0410 (6)	
C9B	0.9301 (3)	0.13854 (16)	0.40664 (17)	0.0481 (6)	
H9C	0.9410	0.0887	0.4704	0.058*	
H9D	1.0055	0.1241	0.3668	0.058*	
C10B	0.7705 (3)	0.14050 (15)	0.37765 (16)	0.0470 (6)	
H10C	0.7559	0.0806	0.3781	0.056*	
H10D	0.6948	0.1477	0.4217	0.056*	
C11B	0.6746 (3)	0.21266 (17)	0.21564 (17)	0.0452 (6)	
H11B	0.6374	0.1541	0.2301	0.054*	
C12B	0.6552 (3)	0.28971 (18)	0.12756 (17)	0.0466 (6)	
C13B	0.7156 (3)	0.37574 (17)	0.10419 (16)	0.0485 (6)	
H13B	0.7063	0.4270	0.0447	0.058*	
C14B	0.7905 (3)	0.38470 (16)	0.17052 (16)	0.0450 (6)	
H14B	0.8327	0.4424	0.1543	0.054*	
C15B	0.7752 (3)	0.48667 (15)	0.29184 (15)	0.0413 (6)	
C16B	0.8150 (3)	0.57949 (16)	0.26625 (16)	0.0484 (6)	
C17B	0.8906 (3)	0.40605 (15)	0.33414 (15)	0.0394 (5)	
C18B	1.0014 (3)	0.41609 (16)	0.38601 (16)	0.0415 (6)	
C19B	0.8840 (2)	0.31768 (15)	0.33252 (15)	0.0371 (5)	
C20B	0.9591 (2)	0.23375 (15)	0.39936 (15)	0.0391 (5)	
C21B	0.8044 (2)	0.31013 (15)	0.26054 (15)	0.0370 (5)	
C22B	0.7482 (2)	0.22162 (15)	0.28218 (15)	0.0383 (5)	
C23B	0.4664 (5)	0.7987 (2)	0.1717 (3)	0.1213 (15)	
H23D	0.3733	0.8387	0.1548	0.182*	
H23E	0.5366	0.8175	0.1178	0.182*	
H23F	0.5123	0.8055	0.2192	0.182*	
C24B	0.5497 (4)	0.3489 (2)	−0.0203 (2)	0.0901 (10)	
H24D	0.4958	0.3272	−0.0547	0.135*	
H24E	0.6450	0.3737	−0.0537	0.135*	
H24F	0.4879	0.3984	−0.0129	0.135*	
C25B	1.1926 (3)	0.3186 (2)	0.51467 (18)	0.0534 (7)	
C26B	1.1451 (3)	0.17239 (19)	0.53251 (17)	0.0498 (6)	

### Atomic displacement parameters (Å^2^)

**Table:**

	*U*^11^	*U*^22^	*U*^33^	*U*^12^	*U*^13^	*U*^23^
O1	0.0713 (12)	0.0406 (10)	0.0656 (12)	−0.0097 (9)	−0.0121 (10)	−0.0208 (9)
O2	0.0628 (12)	0.1206 (17)	0.0705 (12)	0.0183 (11)	−0.0239 (10)	−0.0726 (13)
O3	0.0602 (11)	0.0715 (13)	0.0662 (12)	−0.0122 (9)	−0.0033 (10)	−0.0477 (10)
O4	0.0737 (12)	0.0499 (11)	0.0646 (12)	−0.0148 (9)	0.0019 (10)	−0.0343 (10)
O5	0.1114 (17)	0.0406 (12)	0.0715 (14)	−0.0087 (11)	−0.0069 (12)	−0.0163 (11)
C1	0.0413 (13)	0.0467 (15)	0.0422 (14)	−0.0038 (11)	−0.0092 (11)	−0.0215 (12)
C2	0.0435 (14)	0.0499 (16)	0.0515 (15)	−0.0100 (11)	−0.0077 (12)	−0.0256 (13)
C3	0.0514 (15)	0.0410 (14)	0.0443 (14)	−0.0099 (11)	−0.0008 (12)	−0.0214 (12)
C4	0.0509 (15)	0.0418 (14)	0.0398 (13)	−0.0001 (11)	−0.0115 (11)	−0.0177 (12)
C5	0.0413 (13)	0.0473 (14)	0.0422 (13)	−0.0033 (11)	−0.0116 (11)	−0.0219 (12)
C6	0.0473 (14)	0.0490 (15)	0.0392 (13)	−0.0090 (11)	−0.0072 (11)	−0.0244 (12)
C7	0.0402 (13)	0.0441 (14)	0.0391 (13)	−0.0093 (10)	0.0029 (10)	−0.0220 (12)
C8	0.0488 (14)	0.0385 (14)	0.0451 (15)	−0.0076 (11)	0.0059 (11)	−0.0200 (12)
C9	0.0667 (18)	0.0458 (15)	0.0484 (15)	0.0065 (13)	−0.0072 (13)	−0.0181 (13)
C10	0.0546 (16)	0.0589 (17)	0.0472 (15)	0.0137 (13)	−0.0142 (12)	−0.0220 (13)
C11	0.0430 (14)	0.0713 (18)	0.0426 (14)	0.0054 (13)	−0.0138 (11)	−0.0277 (14)
C12	0.0417 (14)	0.0731 (18)	0.0449 (14)	−0.0003 (13)	−0.0078 (12)	−0.0356 (14)
C13	0.0468 (15)	0.0594 (16)	0.0494 (15)	0.0053 (12)	−0.0068 (12)	−0.0336 (13)
C14	0.0400 (13)	0.0516 (15)	0.0429 (14)	0.0037 (11)	−0.0106 (11)	−0.0242 (12)
C15	0.0391 (13)	0.0423 (14)	0.0349 (12)	−0.0025 (10)	−0.0059 (10)	−0.0205 (11)
C16	0.0389 (13)	0.0447 (14)	0.0353 (13)	−0.0045 (10)	−0.0037 (10)	−0.0217 (11)
C17	0.0331 (12)	0.0415 (14)	0.0401 (13)	−0.0020 (10)	−0.0021 (10)	−0.0217 (11)
C18	0.0365 (12)	0.0433 (14)	0.0376 (13)	−0.0053 (10)	−0.0008 (10)	−0.0216 (11)
C19	0.0349 (12)	0.0445 (14)	0.0365 (13)	−0.0002 (10)	−0.0048 (10)	−0.0197 (11)
C20	0.0477 (14)	0.0407 (14)	0.0390 (13)	−0.0011 (11)	−0.0019 (11)	−0.0169 (11)
C21	0.0405 (13)	0.0451 (14)	0.0346 (13)	−0.0010 (11)	−0.0052 (10)	−0.0189 (11)
C22	0.0407 (13)	0.0520 (15)	0.0377 (13)	0.0028 (11)	−0.0064 (11)	−0.0197 (12)
C23	0.088 (2)	0.0438 (16)	0.0642 (18)	−0.0012 (15)	−0.0185 (16)	−0.0187 (14)
C24	0.068 (2)	0.172 (4)	0.115 (3)	0.028 (2)	−0.0244 (19)	−0.119 (3)
C25	0.0458 (15)	0.0491 (16)	0.0543 (16)	−0.0113 (12)	0.0076 (13)	−0.0299 (14)
C26	0.0631 (17)	0.0448 (17)	0.0548 (17)	−0.0122 (13)	0.0091 (14)	−0.0216 (14)
O1B	0.1200 (18)	0.0543 (13)	0.0742 (14)	0.0350 (12)	−0.0089 (13)	−0.0255 (11)
O2B	0.0745 (13)	0.0880 (14)	0.0598 (12)	−0.0165 (10)	−0.0110 (10)	−0.0473 (11)
O3B	0.0811 (14)	0.0756 (14)	0.0928 (15)	0.0114 (11)	−0.0425 (11)	−0.0539 (12)
O4B	0.0601 (11)	0.0601 (12)	0.0571 (11)	0.0153 (9)	−0.0247 (9)	−0.0292 (10)
O5B	0.0698 (13)	0.0470 (12)	0.0712 (13)	0.0180 (9)	−0.0205 (10)	−0.0195 (10)
C1B	0.0567 (16)	0.0393 (14)	0.0456 (14)	0.0044 (12)	−0.0124 (12)	−0.0216 (12)
C2B	0.0574 (16)	0.0521 (17)	0.0498 (15)	0.0121 (13)	−0.0127 (12)	−0.0259 (13)
C3B	0.077 (2)	0.0448 (17)	0.0423 (15)	0.0233 (15)	−0.0115 (14)	−0.0209 (13)
C4B	0.087 (2)	0.0318 (14)	0.0475 (15)	0.0062 (14)	−0.0147 (14)	−0.0181 (12)
C5B	0.085 (2)	0.0359 (15)	0.0640 (18)	−0.0115 (13)	−0.0187 (15)	−0.0198 (13)
C6B	0.0659 (17)	0.0435 (15)	0.0635 (17)	0.0000 (12)	−0.0224 (14)	−0.0296 (14)
C7B	0.0410 (13)	0.0439 (14)	0.0420 (13)	0.0017 (11)	−0.0055 (11)	−0.0231 (12)
C8B	0.0364 (13)	0.0408 (14)	0.0418 (13)	0.0040 (10)	−0.0039 (11)	−0.0170 (11)
C9B	0.0474 (15)	0.0333 (13)	0.0571 (16)	−0.0012 (11)	−0.0036 (12)	−0.0159 (12)
C10B	0.0506 (15)	0.0328 (13)	0.0555 (15)	−0.0050 (11)	−0.0046 (12)	−0.0183 (12)
C11B	0.0449 (14)	0.0441 (15)	0.0557 (16)	−0.0093 (11)	0.0047 (12)	−0.0311 (13)
C12B	0.0448 (14)	0.0596 (17)	0.0492 (15)	−0.0032 (12)	0.0000 (12)	−0.0371 (14)
C13B	0.0572 (16)	0.0500 (16)	0.0398 (14)	−0.0022 (12)	−0.0044 (12)	−0.0221 (12)
C14B	0.0573 (15)	0.0356 (13)	0.0432 (14)	−0.0049 (11)	−0.0048 (12)	−0.0186 (12)
C15B	0.0576 (15)	0.0322 (13)	0.0359 (13)	0.0025 (11)	−0.0117 (11)	−0.0168 (11)
C16B	0.0684 (17)	0.0353 (14)	0.0434 (14)	−0.0009 (12)	−0.0131 (12)	−0.0187 (12)
C17B	0.0476 (14)	0.0344 (13)	0.0363 (13)	−0.0016 (10)	−0.0049 (11)	−0.0163 (11)
C18B	0.0486 (14)	0.0382 (14)	0.0410 (13)	−0.0008 (11)	−0.0070 (11)	−0.0208 (11)
C19B	0.0391 (13)	0.0315 (13)	0.0400 (13)	−0.0030 (10)	−0.0033 (10)	−0.0159 (11)
C20B	0.0376 (13)	0.0339 (13)	0.0429 (13)	−0.0004 (10)	−0.0014 (10)	−0.0162 (11)
C21B	0.0413 (13)	0.0318 (13)	0.0397 (13)	0.0002 (10)	−0.0035 (10)	−0.0186 (11)
C22B	0.0366 (12)	0.0343 (13)	0.0456 (14)	−0.0033 (10)	0.0018 (10)	−0.0206 (11)
C23B	0.149 (4)	0.066 (2)	0.113 (3)	0.026 (2)	0.020 (3)	−0.023 (2)
C24B	0.098 (3)	0.120 (3)	0.065 (2)	−0.009 (2)	−0.0306 (18)	−0.048 (2)
C25B	0.0526 (16)	0.0580 (18)	0.0542 (16)	0.0105 (13)	−0.0146 (13)	−0.0306 (15)
C26B	0.0477 (15)	0.0501 (17)	0.0495 (15)	0.0109 (13)	−0.0086 (12)	−0.0230 (14)

### Geometric parameters (Å, º)

**Table:**

O1—C3	1.362 (3)	O1B—C23B	1.352 (4)
O1—C23	1.429 (3)	O1B—C3B	1.379 (3)
O2—C12	1.368 (3)	O2B—C12B	1.364 (3)
O2—C24	1.414 (3)	O2B—C24B	1.418 (3)
O3—C25	1.192 (3)	O3B—C25B	1.193 (3)
O4—C25	1.399 (3)	O4B—C26B	1.397 (3)
O4—C26	1.401 (3)	O4B—C25B	1.399 (3)
O5—C26	1.193 (3)	O5B—C26B	1.194 (3)
C1—C2	1.374 (3)	C1B—C2B	1.367 (3)
C1—C15	1.399 (3)	C1B—C15B	1.398 (3)
C1—H1	0.9300	C1B—H1B	0.9300
C2—C3	1.386 (3)	C2B—C3B	1.384 (3)
C2—H2	0.9300	C2B—H2B	0.9300
C3—C4	1.387 (3)	C3B—C4B	1.376 (4)
C4—C16	1.391 (3)	C4B—C16B	1.386 (3)
C4—H4	0.9300	C4B—H4B	0.9300
C5—C16	1.506 (3)	C5B—C16B	1.497 (3)
C5—C6	1.518 (3)	C5B—C6B	1.521 (3)
C5—H5A	0.9700	C5B—H5C	0.9700
C5—H5B	0.9700	C5B—H5D	0.9700
C6—C18	1.498 (3)	C6B—C18B	1.508 (3)
C6—H6A	0.9700	C6B—H6C	0.9700
C6—H6B	0.9700	C6B—H6D	0.9700
C7—C18	1.378 (3)	C7B—C18B	1.379 (3)
C7—C8	1.387 (3)	C7B—C8B	1.391 (3)
C7—C25	1.478 (3)	C7B—C25B	1.469 (3)
C8—C20	1.395 (3)	C8B—C20B	1.385 (3)
C8—C26	1.474 (3)	C8B—C26B	1.464 (3)
C9—C20	1.505 (3)	C9B—C20B	1.506 (3)
C9—C10	1.518 (4)	C9B—C10B	1.511 (3)
C9—H9A	0.9700	C9B—H9C	0.9700
C9—H9B	0.9700	C9B—H9D	0.9700
C10—C22	1.501 (3)	C10B—C22B	1.493 (3)
C10—H10A	0.9700	C10B—H10C	0.9700
C10—H10B	0.9700	C10B—H10D	0.9700
C11—C12	1.379 (3)	C11B—C22B	1.382 (3)
C11—C22	1.388 (3)	C11B—C12B	1.389 (3)
C11—H11	0.9300	C11B—H11B	0.9300
C12—C13	1.378 (3)	C12B—C13B	1.380 (3)
C13—C14	1.383 (3)	C13B—C14B	1.385 (3)
C13—H13	0.9300	C13B—H13B	0.9300
C14—C21	1.389 (3)	C14B—C21B	1.389 (3)
C14—H14	0.9300	C14B—H14B	0.9300
C15—C16	1.399 (3)	C15B—C16B	1.400 (3)
C15—C17	1.480 (3)	C15B—C17B	1.478 (3)
C17—C18	1.421 (3)	C17B—C18B	1.413 (3)
C17—C19	1.427 (3)	C17B—C19B	1.425 (3)
C19—C20	1.410 (3)	C19B—C20B	1.410 (3)
C19—C21	1.488 (3)	C19B—C21B	1.478 (3)
C21—C22	1.401 (3)	C21B—C22B	1.406 (3)
C23—H23A	0.9600	C23B—H23D	0.9600
C23—H23B	0.9600	C23B—H23E	0.9600
C23—H23C	0.9600	C23B—H23F	0.9600
C24—H24A	0.9600	C24B—H24D	0.9600
C24—H24B	0.9600	C24B—H24E	0.9600
C24—H24C	0.9600	C24B—H24F	0.9600
			
C3—O1—C23	117.74 (19)	C23B—O1B—C3B	118.2 (3)
C12—O2—C24	118.3 (2)	C12B—O2B—C24B	117.4 (2)
C25—O4—C26	109.91 (19)	C26B—O4B—C25B	109.09 (18)
C2—C1—C15	121.3 (2)	C2B—C1B—C15B	121.2 (2)
C2—C1—H1	119.4	C2B—C1B—H1B	119.4
C15—C1—H1	119.4	C15B—C1B—H1B	119.4
C1—C2—C3	120.5 (2)	C1B—C2B—C3B	119.7 (3)
C1—C2—H2	119.7	C1B—C2B—H2B	120.2
C3—C2—H2	119.7	C3B—C2B—H2B	120.2
O1—C3—C2	116.1 (2)	C4B—C3B—O1B	125.0 (3)
O1—C3—C4	124.7 (2)	C4B—C3B—C2B	120.5 (2)
C2—C3—C4	119.2 (2)	O1B—C3B—C2B	114.5 (3)
C3—C4—C16	120.4 (2)	C3B—C4B—C16B	120.2 (2)
C3—C4—H4	119.8	C3B—C4B—H4B	119.9
C16—C4—H4	119.8	C16B—C4B—H4B	119.9
C16—C5—C6	109.11 (18)	C16B—C5B—C6B	109.4 (2)
C16—C5—H5A	109.9	C16B—C5B—H5C	109.8
C6—C5—H5A	109.9	C6B—C5B—H5C	109.8
C16—C5—H5B	109.9	C16B—C5B—H5D	109.8
C6—C5—H5B	109.9	C6B—C5B—H5D	109.8
H5A—C5—H5B	108.3	H5C—C5B—H5D	108.2
C18—C6—C5	110.95 (18)	C18B—C6B—C5B	110.5 (2)
C18—C6—H6A	109.4	C18B—C6B—H6C	109.6
C5—C6—H6A	109.4	C5B—C6B—H6C	109.6
C18—C6—H6B	109.4	C18B—C6B—H6D	109.6
C5—C6—H6B	109.4	C5B—C6B—H6D	109.6
H6A—C6—H6B	108.0	H6C—C6B—H6D	108.1
C18—C7—C8	122.4 (2)	C18B—C7B—C8B	122.1 (2)
C18—C7—C25	129.9 (2)	C18B—C7B—C25B	130.5 (2)
C8—C7—C25	107.7 (2)	C8B—C7B—C25B	107.4 (2)
C7—C8—C20	121.9 (2)	C20B—C8B—C7B	122.0 (2)
C7—C8—C26	108.0 (2)	C20B—C8B—C26B	130.1 (2)
C20—C8—C26	130.0 (2)	C7B—C8B—C26B	107.8 (2)
C20—C9—C10	109.8 (2)	C20B—C9B—C10B	110.35 (18)
C20—C9—H9A	109.7	C20B—C9B—H9C	109.6
C10—C9—H9A	109.7	C10B—C9B—H9C	109.6
C20—C9—H9B	109.7	C20B—C9B—H9D	109.6
C10—C9—H9B	109.7	C10B—C9B—H9D	109.6
H9A—C9—H9B	108.2	H9C—C9B—H9D	108.1
C22—C10—C9	108.9 (2)	C22B—C10B—C9B	110.17 (19)
C22—C10—H10A	109.9	C22B—C10B—H10C	109.6
C9—C10—H10A	109.9	C9B—C10B—H10C	109.6
C22—C10—H10B	109.9	C22B—C10B—H10D	109.6
C9—C10—H10B	109.9	C9B—C10B—H10D	109.6
H10A—C10—H10B	108.3	H10C—C10B—H10D	108.1
C12—C11—C22	121.0 (2)	C22B—C11B—C12B	121.1 (2)
C12—C11—H11	119.5	C22B—C11B—H11B	119.5
C22—C11—H11	119.5	C12B—C11B—H11B	119.5
O2—C12—C13	124.7 (2)	O2B—C12B—C13B	125.0 (2)
O2—C12—C11	115.5 (2)	O2B—C12B—C11B	115.1 (2)
C13—C12—C11	119.9 (2)	C13B—C12B—C11B	119.9 (2)
C12—C13—C14	119.5 (2)	C12B—C13B—C14B	119.1 (2)
C12—C13—H13	120.2	C12B—C13B—H13B	120.4
C14—C13—H13	120.2	C14B—C13B—H13B	120.4
C13—C14—C21	121.7 (2)	C13B—C14B—C21B	122.0 (2)
C13—C14—H14	119.2	C13B—C14B—H14B	119.0
C21—C14—H14	119.2	C21B—C14B—H14B	119.0
C16—C15—C1	117.8 (2)	C1B—C15B—C16B	118.5 (2)
C16—C15—C17	119.52 (19)	C1B—C15B—C17B	121.6 (2)
C1—C15—C17	122.51 (19)	C16B—C15B—C17B	119.1 (2)
C4—C16—C15	120.6 (2)	C4B—C16B—C15B	119.8 (2)
C4—C16—C5	121.27 (19)	C4B—C16B—C5B	123.0 (2)
C15—C16—C5	118.1 (2)	C15B—C16B—C5B	117.1 (2)
C18—C17—C19	119.55 (19)	C18B—C17B—C19B	120.04 (19)
C18—C17—C15	116.29 (18)	C18B—C17B—C15B	116.69 (19)
C19—C17—C15	124.11 (19)	C19B—C17B—C15B	122.9 (2)
C7—C18—C17	117.29 (19)	C7B—C18B—C17B	117.0 (2)
C7—C18—C6	123.47 (19)	C7B—C18B—C6B	123.7 (2)
C17—C18—C6	119.24 (19)	C17B—C18B—C6B	119.31 (19)
C20—C19—C17	120.94 (19)	C20B—C19B—C17B	120.2 (2)
C20—C19—C21	115.84 (19)	C20B—C19B—C21B	117.42 (19)
C17—C19—C21	123.10 (19)	C17B—C19B—C21B	122.36 (19)
C8—C20—C19	116.6 (2)	C8B—C20B—C19B	116.9 (2)
C8—C20—C9	123.6 (2)	C8B—C20B—C9B	123.6 (2)
C19—C20—C9	119.8 (2)	C19B—C20B—C9B	119.4 (2)
C14—C21—C22	118.2 (2)	C14B—C21B—C22B	118.2 (2)
C14—C21—C19	122.64 (19)	C14B—C21B—C19B	123.1 (2)
C22—C21—C19	118.9 (2)	C22B—C21B—C19B	118.56 (19)
C11—C22—C21	119.6 (2)	C11B—C22B—C21B	119.6 (2)
C11—C22—C10	122.5 (2)	C11B—C22B—C10B	122.5 (2)
C21—C22—C10	117.8 (2)	C21B—C22B—C10B	117.9 (2)
O1—C23—H23A	109.5	O1B—C23B—H23D	109.5
O1—C23—H23B	109.5	O1B—C23B—H23E	109.5
H23A—C23—H23B	109.5	H23D—C23B—H23E	109.5
O1—C23—H23C	109.5	O1B—C23B—H23F	109.5
H23A—C23—H23C	109.5	H23D—C23B—H23F	109.5
H23B—C23—H23C	109.5	H23E—C23B—H23F	109.5
O2—C24—H24A	109.5	O2B—C24B—H24D	109.5
O2—C24—H24B	109.5	O2B—C24B—H24E	109.5
H24A—C24—H24B	109.5	H24D—C24B—H24E	109.5
O2—C24—H24C	109.5	O2B—C24B—H24F	109.5
H24A—C24—H24C	109.5	H24D—C24B—H24F	109.5
H24B—C24—H24C	109.5	H24E—C24B—H24F	109.5
O3—C25—O4	120.1 (2)	O3B—C25B—O4B	120.1 (2)
O3—C25—C7	132.6 (2)	O3B—C25B—C7B	132.1 (2)
O4—C25—C7	107.2 (2)	O4B—C25B—C7B	107.8 (2)
O5—C26—O4	120.5 (2)	O5B—C26B—O4B	119.8 (2)
O5—C26—C8	132.3 (3)	O5B—C26B—C8B	132.3 (2)
O4—C26—C8	107.2 (2)	O4B—C26B—C8B	107.9 (2)
			
C15—C1—C2—C3	−0.6 (3)	C15B—C1B—C2B—C3B	−0.2 (4)
C23—O1—C3—C2	170.0 (2)	C23B—O1B—C3B—C4B	4.1 (4)
C23—O1—C3—C4	−10.5 (3)	C23B—O1B—C3B—C2B	−176.9 (3)
C1—C2—C3—O1	−177.3 (2)	C1B—C2B—C3B—C4B	−1.0 (4)
C1—C2—C3—C4	3.2 (3)	C1B—C2B—C3B—O1B	179.9 (2)
O1—C3—C4—C16	178.8 (2)	O1B—C3B—C4B—C16B	179.1 (2)
C2—C3—C4—C16	−1.8 (3)	C2B—C3B—C4B—C16B	0.1 (4)
C16—C5—C6—C18	−55.7 (2)	C16B—C5B—C6B—C18B	55.4 (3)
C18—C7—C8—C20	−5.4 (3)	C18B—C7B—C8B—C20B	6.9 (3)
C25—C7—C8—C20	175.5 (2)	C25B—C7B—C8B—C20B	−176.3 (2)
C18—C7—C8—C26	177.6 (2)	C18B—C7B—C8B—C26B	−174.9 (2)
C25—C7—C8—C26	−1.5 (2)	C25B—C7B—C8B—C26B	1.8 (2)
C20—C9—C10—C22	−57.2 (3)	C20B—C9B—C10B—C22B	55.0 (3)
C24—O2—C12—C13	−2.9 (4)	C24B—O2B—C12B—C13B	−5.7 (4)
C24—O2—C12—C11	175.0 (3)	C24B—O2B—C12B—C11B	176.7 (2)
C22—C11—C12—O2	−177.7 (2)	C22B—C11B—C12B—O2B	−179.6 (2)
C22—C11—C12—C13	0.3 (4)	C22B—C11B—C12B—C13B	2.7 (3)
O2—C12—C13—C14	179.0 (2)	O2B—C12B—C13B—C14B	−179.7 (2)
C11—C12—C13—C14	1.2 (4)	C11B—C12B—C13B—C14B	−2.2 (3)
C12—C13—C14—C21	0.1 (4)	C12B—C13B—C14B—C21B	−1.1 (3)
C2—C1—C15—C16	−3.4 (3)	C2B—C1B—C15B—C16B	2.2 (3)
C2—C1—C15—C17	−178.9 (2)	C2B—C1B—C15B—C17B	172.3 (2)
C3—C4—C16—C15	−2.3 (3)	C3B—C4B—C16B—C15B	2.0 (4)
C3—C4—C16—C5	175.5 (2)	C3B—C4B—C16B—C5B	−174.2 (2)
C1—C15—C16—C4	4.8 (3)	C1B—C15B—C16B—C4B	−3.1 (3)
C17—C15—C16—C4	−179.51 (19)	C17B—C15B—C16B—C4B	−173.4 (2)
C1—C15—C16—C5	−173.1 (2)	C1B—C15B—C16B—C5B	173.4 (2)
C17—C15—C16—C5	2.6 (3)	C17B—C15B—C16B—C5B	3.0 (3)
C6—C5—C16—C4	−138.3 (2)	C6B—C5B—C16B—C4B	131.1 (2)
C6—C5—C16—C15	39.5 (3)	C6B—C5B—C16B—C15B	−45.2 (3)
C16—C15—C17—C18	−29.0 (3)	C1B—C15B—C17B—C18B	−140.7 (2)
C1—C15—C17—C18	146.5 (2)	C16B—C15B—C17B—C18B	29.3 (3)
C16—C15—C17—C19	153.7 (2)	C1B—C15B—C17B—C19B	32.6 (3)
C1—C15—C17—C19	−30.8 (3)	C16B—C15B—C17B—C19B	−157.3 (2)
C8—C7—C18—C17	2.0 (3)	C8B—C7B—C18B—C17B	0.7 (3)
C25—C7—C18—C17	−179.2 (2)	C25B—C7B—C18B—C17B	−175.2 (2)
C8—C7—C18—C6	−178.1 (2)	C8B—C7B—C18B—C6B	178.5 (2)
C25—C7—C18—C6	0.7 (4)	C25B—C7B—C18B—C6B	2.6 (4)
C19—C17—C18—C7	7.2 (3)	C19B—C17B—C18B—C7B	−11.8 (3)
C15—C17—C18—C7	−170.15 (19)	C15B—C17B—C18B—C7B	161.8 (2)
C19—C17—C18—C6	−172.72 (19)	C19B—C17B—C18B—C6B	170.2 (2)
C15—C17—C18—C6	9.9 (3)	C15B—C17B—C18B—C6B	−16.2 (3)
C5—C6—C18—C7	−147.6 (2)	C5B—C6B—C18B—C7B	156.3 (2)
C5—C6—C18—C17	32.4 (3)	C5B—C6B—C18B—C17B	−25.9 (3)
C18—C17—C19—C20	−13.5 (3)	C18B—C17B—C19B—C20B	15.9 (3)
C15—C17—C19—C20	163.7 (2)	C15B—C17B—C19B—C20B	−157.3 (2)
C18—C17—C19—C21	162.52 (19)	C18B—C17B—C19B—C21B	−162.1 (2)
C15—C17—C19—C21	−20.3 (3)	C15B—C17B—C19B—C21B	24.8 (3)
C7—C8—C20—C19	−0.6 (3)	C7B—C8B—C20B—C19B	−2.9 (3)
C26—C8—C20—C19	175.6 (2)	C26B—C8B—C20B—C19B	179.4 (2)
C7—C8—C20—C9	−179.3 (2)	C7B—C8B—C20B—C9B	178.8 (2)
C26—C8—C20—C9	−3.0 (4)	C26B—C8B—C20B—C9B	1.1 (4)
C17—C19—C20—C8	9.9 (3)	C17B—C19B—C20B—C8B	−8.3 (3)
C21—C19—C20—C8	−166.4 (2)	C21B—C19B—C20B—C8B	169.83 (19)
C17—C19—C20—C9	−171.4 (2)	C17B—C19B—C20B—C9B	170.1 (2)
C21—C19—C20—C9	12.3 (3)	C21B—C19B—C20B—C9B	−11.8 (3)
C10—C9—C20—C8	−150.1 (2)	C10B—C9B—C20B—C8B	149.0 (2)
C10—C9—C20—C19	31.3 (3)	C10B—C9B—C20B—C19B	−29.3 (3)
C13—C14—C21—C22	−2.9 (3)	C13B—C14B—C21B—C22B	3.8 (3)
C13—C14—C21—C19	−176.9 (2)	C13B—C14B—C21B—C19B	179.8 (2)
C20—C19—C21—C14	143.3 (2)	C20B—C19B—C21B—C14B	−148.4 (2)
C17—C19—C21—C14	−32.9 (3)	C17B—C19B—C21B—C14B	29.6 (3)
C20—C19—C21—C22	−30.7 (3)	C20B—C19B—C21B—C22B	27.5 (3)
C17—C19—C21—C22	153.1 (2)	C17B—C19B—C21B—C22B	−154.4 (2)
C12—C11—C22—C21	−3.2 (4)	C12B—C11B—C22B—C21B	0.1 (3)
C12—C11—C22—C10	173.5 (2)	C12B—C11B—C22B—C10B	179.7 (2)
C14—C21—C22—C11	4.4 (3)	C14B—C21B—C22B—C11B	−3.3 (3)
C19—C21—C22—C11	178.6 (2)	C19B—C21B—C22B—C11B	−179.41 (19)
C14—C21—C22—C10	−172.5 (2)	C14B—C21B—C22B—C10B	177.1 (2)
C19—C21—C22—C10	1.8 (3)	C19B—C21B—C22B—C10B	0.9 (3)
C9—C10—C22—C11	−134.6 (2)	C9B—C10B—C22B—C11B	138.1 (2)
C9—C10—C22—C21	42.2 (3)	C9B—C10B—C22B—C21B	−42.3 (3)
C26—O4—C25—O3	−179.8 (2)	C26B—O4B—C25B—O3B	−178.2 (2)
C26—O4—C25—C7	0.0 (2)	C26B—O4B—C25B—C7B	1.8 (3)
C18—C7—C25—O3	1.8 (4)	C18B—C7B—C25B—O3B	−5.9 (5)
C8—C7—C25—O3	−179.3 (3)	C8B—C7B—C25B—O3B	177.7 (3)
C18—C7—C25—O4	−178.0 (2)	C18B—C7B—C25B—O4B	174.2 (2)
C8—C7—C25—O4	0.9 (2)	C8B—C7B—C25B—O4B	−2.2 (3)
C25—O4—C26—O5	−179.9 (2)	C25B—O4B—C26B—O5B	179.5 (2)
C25—O4—C26—C8	−0.9 (2)	C25B—O4B—C26B—C8B	−0.7 (3)
C7—C8—C26—O5	−179.7 (3)	C20B—C8B—C26B—O5B	−3.1 (5)
C20—C8—C26—O5	3.6 (5)	C7B—C8B—C26B—O5B	179.0 (3)
C7—C8—C26—O4	1.5 (3)	C20B—C8B—C26B—O4B	177.2 (2)
C20—C8—C26—O4	−175.2 (2)	C7B—C8B—C26B—O4B	−0.8 (3)

### Hydrogen-bond geometry (Å, º)

**Table:**

*D*—H···*A*	*D*—H	H···*A*	*D*···*A*	*D*—H···*A*
C1—H1···O2^i^	0.93	2.58	3.304 (3)	135
C23—H23*A*···O3*B*^ii^	0.96	2.71	3.666 (3)	177
C6*B*—H6*D*···O3*B*^iii^	0.97	2.68	3.547 (3)	149
C10*B*—H10*C*···O5*B*^iv^	0.97	2.44	3.297 (3)	147
C13*B*—H13*B*···O5^v^	0.93	2.56	3.408 (3)	152
C24*B*—H24*D*···O1*B*^v^	0.96	2.74	3.491 (4)	136

Symmetry codes: (i) −*x*, −*y*, −*z*; (ii) *x*−1, *y*−1, *z*; (iii) −*x*+2, −*y*+1, −*z*+1; (iv) −*x*+2, −*y*, −*z*+1; (v) −*x*+1, −*y*+1, −*z*.
